# Richmond Academy of Medicine and Surgery

**Published:** 1893-04

**Authors:** J. F. Woodward

**Affiliations:** Reporter; Richmond, Va.


### *SOCIETY REPORTS.*

### RICHMOND ACADEMY OF MEDICINE AND SURGERY.

RICHMOND, VA., February 14, 1893.

REFLEX VESICAL IRRITATION.

DR. JNO. N. UPSHUR read a very instructive paper on this subject, basing his remarks chiefly upon reflexes more commonly found in women, and dwelt especially upon those relative to true uterine lesions, where a neurotic element existed.

MR. BLAIR thought the causes of these reflexes very obscure, and suggested that the often spoken of failures in these cases were due to an oversight, and as an analytical chemist he presented some interesting ideas concerning the deposits usually found in these and other bladder troubles, referring more especially to phospatic urine, and claimed that to sterilize urine an alkaline condition was necessary rather than an acid. Thought the deposits often found on the mucous membrane of bladder were as a rule phosphatic and not uric acid, and that the alkaline condition more often produced cystitis than the acid. He always found the phosphatic urine of an offensive odor, and phosphatenia generally indicated depression and debility.

DR. EDWARDS remarked that this subject was worthy of much study, and was decidedly in favor of giving therapeusis a trial rather than rush into surgery, as in his mind very few of the cases ever reached such a grave state as to require the knife. That lesions about neck of womb more often cause bladder reflex and gave as personal experience the boracic acid treatment, viz.: pack vagina with boracic acid, say one ounce; let it remain for three or four days and then repack or use iodol locally. This treatment, though simple, he had seen act like a charm in many cases. Also suggested that suspicious cases of gonorrhœa should not be neglected as a cause in these reflexes.

### DOUBTFUL APPENDICITIS.

DR. W. W. PARKER reported a case, child six years old, with what he at first thought obstruction of bowels—nausea, vomiting and constipation, with some colicky pains uncontrollable by usual remedies. Pulse feeble and quick, skin bathed in clammy sweat. In about forty-eight hours, however, the little patient began to improve, and was thought out of danger. About ten days later he was called again, when all the former symptoms were exaggerated and there was strong suspicion of appendicitis. Abdomen distended in right iliac region and collapse imminent. The perineum was opened and a large quantity of pus let out, flushed the cavity and awaited the result. The child died shortly afterward. The doctor wanted to know whether or not he should have continued his operations, as the first incision was only exploratory.

DR. HUGH M. TAYLOR thought the case reported by Dr. Parker was one of appendicitis, acute for the first few days, then subacute or quiescent and again becoming acute, and finally ending in suppuration, peritonitis and death. The result showed that it was a case for operative interference with fair prospects of success in the early stages of the disease, but a much lessened chance if the operation is delayed. No problem in the practice of surgery was more difficult of solution, he thought, than that of ascertaining when to operate in appendicitis. With no pathognomonic symptoms we had to judge each case upon its own merits, and it seemed to him that every case he saw presented some unique feature. One thing was beyond all question settled, and that was that operative interference is urgently called for in every case as soon as the chain of symptoms point to the existence of suppuration.

DR. UPSHUR thought it well to distinguish between idiopathic and traumatic appendicitis, as the latter always called for surgery, while the former generally did well under drugs. There was doubt in his mind when to operate, as salines, leeches and poultices had done well in his hands, while much swelling with sense of throbbing and collapse, other symptoms exaggerated, seemed to suggest the use of the knife.

DR. MICHAUX referred to fifteen cases that had come under his care with complete recovery without the use of the knife. This was, in his mind, good evidence that conservatism in these cases would in a major portion of such cases prove successful. He wished to emphasize the benefits to be derived from drugs and prompt attention.

DR. JAS. N. ELLIS thought he had had occasion in last ten days to utilize the practical force of what had just been said as to where and how to operate in appendicitis, and reported the case of a young lady, aged 22, whom he was called to see about two weeks ago. She exhibited all the symptoms we have learned to associate with appendicitis, viz.: chill, nausea and vomiting, constipation, slight fever, rigidity of abdominal muscles of the affected side, elevation of right thigh, irritable pulse, pain and tenderness in right iliac fossa referred to by McBurney. He controlled pain with hypodermic of morphia, ordered teaspoonful of saturated solution of sulphate magnesia every hour, and applied a blister over cæcal region. The magnesia was followed by two ounces castor oil. Subsequently poultices were applied to blistered surface. The constipation continued; any further attempt at purging seemed to increase the pain. Contemplating an operation, he called in consulation Dr. Isaiah White, who confirmed diagnosis, but advised a little more time. During the next four days, while the doctor was reproaching himself for jeopardizing life of the patient by not operating, the symptoms began to change, and in seven days she was out of danger. With such an experience he was inclined to think that so grave an operation is hardly justifiable until there are very conclusive evidences of pus formation.

J. F. WOODWARD, M. D., Reporter.

